# A missing, but essential, platform for multidisciplinary scientific discussion: understanding the ‘elephant’

**DOI:** 10.2144/fsoa-2020-0188

**Published:** 2020-11-30

**Authors:** Morteza Mahmoudi

**Affiliations:** 1 Department of Radiology & Precision Health Program, Michigan State University, East Lansing, MI 48824, USA

**Keywords:** biology, chemistry, human behavior, multidiscipline, physics, scientific discussion, the theory of everything

The current COVID-19 pandemic has unprecedently attracted the focus of research in many branches of science. Although many individual fields (both highly relevant ones such as medicine [1] and perhaps less relevant ones such as physics [2]) are intensively working to address this pandemic, the scientific community lacks effective crossdisciplinary discussion. The parable of the Blind Men and the Elephant [3] might be a good metaphor for our current situation, and point the way towards a constructive discussion/collaboration across different disciplines that may better facilitate our deep understanding of major scientific and humanities issues, including the COVID-19 pandemic. The story describes a group of blind men who touch different parts of an elephant for the first time and compare their experiences [3]. Each man provides accurate, but only partial, information, completely different from the other men. They cannot synthesize an accurate picture of an elephant that encompasses their distinct experiences of the trunk, the ear, the leg, the side, the tail and a tusk. In other words, working exclusively in our own siloed fields of study constricts what we can see and achieve. Instead, combining our different individual lenses/experiences/expertise in a constructive and receptive discussion platform may enable the scientific community to solve urgent and/or important problems in a more effective and timely manner.

A cursory analysis of the fast-growing publications in the field of COVID-19 demonstrates the involvement of many scientific fields, but such extensive efforts lack adequate collaboration or multidisciplinary coordination. Due to prior efforts in the creation of dual degrees (e.g., MD-PhD programs [4]), there is a smaller knowledge gap between engineers, chemists and physicians (compared with physicians and other fields including physics), which do facilitate effective collaborative discussions for solving clinical issues. However, the lack of such well-established programs across other scientific fields (e.g., physics and medicine) can hinder if not actually prevent scientific progress that might adequately address such multi-faceted problems as COVID-19 at their roots and arrive at the best possible diagnostic and therapeutic solutions. For example, effective collaboration/discussion between physicists and physicians could help arrive at more accurate calculations regarding how and to what extent droplets of various sizes containing SARS-CoV-2 move and remain in the air. That could be of enormous help in: better defining the policies and guidelines for appropriate social distancing and the minimum required features of facemasks as well as; earlier prediction of possible airborne nature of the virus [5].

The same strategy might be valid for other major scientific issues, even it may seems to be very specific to some fields such as the search for validation or rejection of the ‘theory of everything,’ or a unified field theory that relies mainly on the expertise of theoretical physicists and mathematicians. As the theory of everything enables the scientific community to explain everything in the universe (including super-emergent species like humans and their behavior), one might expect to see the participation of other scientific fields/branches (e.g., chemistry, biology, neuroscience and human behavioral sciences) to propose and develop other theories on the same scale. However, what we instead witness today are huge efforts by scientists in a few scientific fields including theoretical physics, while excluding many other scientific fields from discussion in the search for development of the theory of everything and unified field theory (of course if such a reality exist). The few existing collaborations between physicists and physicians demonstrate their great potential in addressing/predicting complex clinical issues. For example, a very recent collaboration between physicists and physicians resulted in the successful application of quantum theories in a framework useful in the fields of decision neuroscience and neuroeconomics [6].

There are several possible approaches to facilitate constructive discussions across areas of scientific expertise. One could be development of new educational programs to bridge knowledge gaps. Establishment of MD-PhD programs is a great example of the success of such educational strategies in improving crosstalk between engineers and physicians, which in turn facilitated the emergence and development of: new multidisciplinary fields including regenerative medicine and bioengineering and; new bio-sciences/-technology to address critical clinical needs. Another strategy could be creating a platform allowing scientists in any field to share their needs/goals/insights with other disciplines in a lay statement. A perfect example is how some leading theoretical physicists including Stephen Hawking [7] and Sean Carroll [8] simplified their concepts to make them accessible to scientists and thinkers with different expertise. Establishment of such a platform may help actually unify the sciences and incorporate insights from diverse scientific fields that may have been neglected. Richard Feynman’s pioneering efforts in connecting biology, chemistry and physics [9], which laid the foundation of nanotechnology and nanomedicine, is a great example of how unifying even a few scientific fields can create novel platforms that address persistent industrial and medical issues. The critical role of quantum biology and definition of life [10] by Erwin Schrödinger is another important example.

I therefore propose the establishment of such a platform by stakeholders including researchers with various expertise, institutions, funding agencies and decision makers to facilitate crosstalk and collaborative discussions among leaders of all scientific fields, encouraging scientists to participate in coordinated efforts, toward the larger goal of creating comprehensive pictures of scientific problems and paving efficient ways to address them.
